# The needle and the damage done: musculoskeletal and vascular complications associated with injected drug use

**DOI:** 10.1186/s13244-020-00903-5

**Published:** 2020-08-26

**Authors:** Francis T. Delaney, Emma Stanley, Ferdia Bolster

**Affiliations:** Department of Radiology, Mater Misericordiae University Hospital, Dublin 7, Ireland

**Keywords:** Injected drug use, Complications, Vascular, Musculoskeletal, Infection

## Abstract

Injected drug use is associated with a wide range of medical complications which are predominantly musculoskeletal and vascular in nature. Illicit drug use is increasing worldwide. Patients with complications of injected drug use often present in a non-specific manner without a reliable clinical history. Musculoskeletal complications are typically infective in aetiology and may vary widely in severity from mild to life-threatening. A multimodal imaging approach is often required for both diagnostic imaging and image-guided sampling. Plain radiographs are often an important initial test, for example in identifying retained needles from injection. Ultrasound and CT play important roles in the assessment of complex soft tissue complications and MRI is the imaging modality of choice for bone and joint disorders. Vascular complications may be venous or arterial in nature and usually occur locally at the injection site. These complications may be related to direct injury to the vessel wall by a needle, or secondary to local infection and inflammation. A multimodal imaging strategy is also often required in the assessment of these vascular complications, typically involving a combination of ultrasound and CT. Familiarity with the multimodal imaging features of the complications related to injected drug use is crucially important as they may be rapidly progressive and life-threatening and require timely diagnosis.

## Key points

- Illicit drug use and the prevalence of associated medical complications is increasing worldwide.
- The clinical presentation of patients with complications arising from injected drug use may vary and be non-specific, making timely recognition of imaging manifestations important.
- Plain radiographs are an important initial imaging investigation in injected drug users.
- CT and ultrasound play a key role in the assessment of soft tissue and vascular complications, depending on their nature and severity.
- MRI is the imaging modality of choice for suspected bone or joint complications.

## Background

Recreational drug use dates back as far as recorded history. It remains highly prevalent today and is associated with a significant health and economic burden. Recent studies attribute 1.3% of all disability-adjusted life years (DALYs) to drug use [1]. Indeed, illicit drug use appears to be increasing with the estimated number of people who have used drugs globally rising from 4.8 to 5.5% between 2009 and 2017 [2]. Almost 1 in 10 adults aged 16–59 years in the UK report illicit drug use in the last year [3].

Abuse of illicit drugs is associated with myriad medical complications which can affect any organ system. These complications depend largely on the type of substance used and the route of administration. They may be related to direct drug toxicity or result indirectly from, for example, infection or risk-taking behaviours. Common routes of administration include nasal or oral ingestion, smoking, subcutaneous injection (“skin popping”) and intravenous injection. Opioids, amphetamines and cocaine are among the most frequently encountered drugs. The manifestations of opioid abuse in particular have been highlighted in recent years with an epidemic of opioid use described in certain countries [4]. Complications of opioid abuse may occur as a result of direct toxicity from high levels of opioids in the body, leading to complications such as respiratory failure or altered consciousness, or relate to administration via injection, including local/distant infection or vascular injury. Furthermore, a disproportionate number of drug-related fatalities are attributable to opioids. In 2017, the use of opioids accounted for 66% of deaths due to drug use disorders [5].

Administration of drugs by injection poses multiple additional health risks when compared to other methods of consumption. The population prevalence of injected drug use is estimated at between 0.09 and 1.3% depending on geographic location [6]. Opioids are typically the most common drug that is injected. Multiple direct and indirect complications can occur as a result of the act of injecting itself, either locally at the injection site or elsewhere in the body. In addition, there is an increased risk of fatal overdose and people who inject drugs (PWID) are affected to a much greater extent by blood-borne infectious diseases such as hepatitis C and human immunodeficiency virus (HIV) which are acquired through the use of shared non-sterile injection equipment [2]. It is estimated that 17.8% of PWID are living with HIV and 52.3% are hepatitis C-antibody positive [6]. This is of particular concern given that 52% of deaths related to drug use are the result of untreated hepatitis C leading to cirrhosis and liver cancer, and 11% are due to HIV/AIDS [6].

Recreational drug users, and PWID in particular, are a complex patient cohort who can be challenging to diagnose and treat due to poor compliance, limited clinical history and complicating co-morbidities. They can often present with non-specific signs and symptoms. The history of drug use is often not forthcoming, such is its illicit nature, creating further diagnostic difficulty. Familiarity with the manifestations of drug use is therefore critically important for both physicians and radiologists, particularly in the emergency department setting and in the case of unusual presentations in young adults. Intravenous drug use may present with abnormalities of any organ system, with musculoskeletal or vascular complications the most common, and may affect multiple parts of the body simultaneously. Therefore, timely recognition of typical imaging features and patterns is imperative.

## Main text

### Soft tissue complications

Infectious complications are the most common reason for inpatient admission in PWID [7]. Soft tissue infection due to intravenous injection with contaminated needles or subcutaneous/intramuscular injection may manifest as a range of disorders of varying severity which include cellulitis, abscess, myositis and necrotising fasciitis. These infections are often polymicrobial and *Staphylococcus aureus* and *Streptococcus pyogenes* are among the most common organisms [7]. Prompt diagnosis and determination of the extent of infection is important as more severe pathologies, such as necrotising fasciitis, require urgent surgical management. In addition to injected drug use, other risk factors for soft tissue infections that should be considered include trauma, recent travel, prior surgery, unprotected sex and immunosuppressive disorders such as diabetes mellitus or cirrhosis.

Cellulitis, an acute infection of the dermis and subcutaneous tissues, is a clinical diagnosis when uncomplicated and is treated with antibiotics and supportive measures [8]. It occurs as a result of direct introduction of pathogens into the dermis. Imaging primarily plays a role where a complication of cellulitis, such as a soft tissue abscess or deep venous thrombosis, is suspected. However, imaging should be considered in all patients with a confirmed history or considerable clinical suspicion of injected drug use due to their higher risk of complications. Soft tissue abscess may develop locally at site of injection or extend to deeper locations due to direct of haematogenous spread. A psoas abscess is a recognised complication of injection into the ipsilateral groin, for example.

**Fig. 1:**
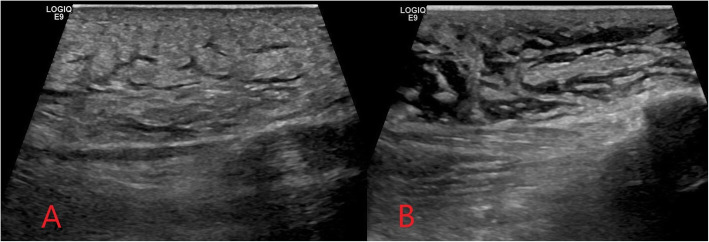
Ultrasound images demonstrating diffuse thickening and increased echogenicity of the subcutaneous tissue (**a**) and cobblestone appearance of the superficial soft tissues (**b**) seen in cellulitis

Plain radiographs may show indirect signs of cellulitis such as soft tissue swelling and loss of fascial planes and can identify radio-opaque retained foreign bodies. Ultrasound is also a useful initial investigation in PWID presenting with soft tissue infection to evaluate for the presence of a subcutaneous abscess or non-radio-opaque foreign body, as well as guiding potential abscess aspiration or therapeutic drainage [8]. Ultrasound features of cellulitis include diffuse thickening and increased echogenicity of subcutaneous tissues and a characteristic “cobblestone” appearance of the subcutaneous fat due to soft tissue oedema [8, 9]. Non-infectious/inflammatory causes of soft tissue oedema such as cardiac failure must be considered as a differential diagnosis. Doppler ultrasound demonstrating diffusely increased flow indicative of hyperaemia (Fig. 1) helps differentiate cellulitis from non-infectious oedema, in addition to clinical history and examination [9]. Computed tomography (CT) in cellulitis demonstrates increased attenuation and stranding of the subcutaneous fat, due to oedema, with overlying skin thickening.

The magnetic resonance imaging (MRI) sequences employed in suspected soft tissue infection will typically include T2, a fluid-sensitive sequence such as short-tau inversion recovery (STIR) and unenhanced and post-contrast T1. Diffusion-weighted imaging (DWI) may be added in certain cases, such as to assess for abscess formation. Fat-saturated T2 or enhanced T1 sequences can be used to improve the delineation of inflammation. A gradient echo sequence may be added in more severe cases with suspected haemorrhage or necrotising fasciitis. In cellulitis, high T2 and STIR signal intensity with corresponding T1 signal intensity of the subcutaneous tissues with overlying skin thickening is seen, and there is enhancement following gadolinium administration [8].

**Fig. 2:**
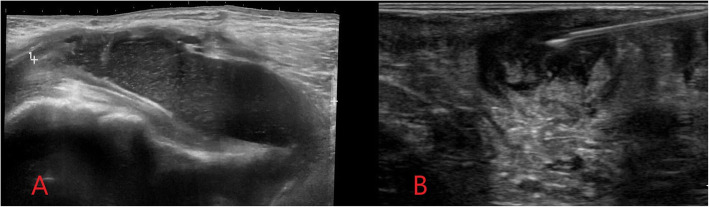
Ultrasound image (**a**) demonstrating a soft tissue groin abscess in an intravenous drug user following subcutaneous injection. An ovoid relatively well-defined anechoic subcutaneous collection is demonstrated with internal echoes consistent with a complex soft tissue abscess. Image (**b**) shows ultrasound-guided aspiration of a soft tissue abscess. A linear hyperechoic needle is visualised with the tip lying within the abscess cavity

Where a soft tissue abscess is present, a relatively well-defined anechoic cavitary subcutaneous or soft tissue collection with peripheral vascularity is typically demonstrated on ultrasound [8]. There may be posterior acoustic enhancement, internal echoes suggestive of complex abscess contents, or septations indicating a loculated abscess (Fig. 2). Complex or loculated abscesses may be difficult to drain percutaneously due to highly viscous contents or difficulty reliably accessing and draining each individual compartment [10]. If imaging-guided percutaneous drainage is not possible, surgical incision and drainage is usually required for abscesses larger than 3 cm [10].

**Fig. 3:**
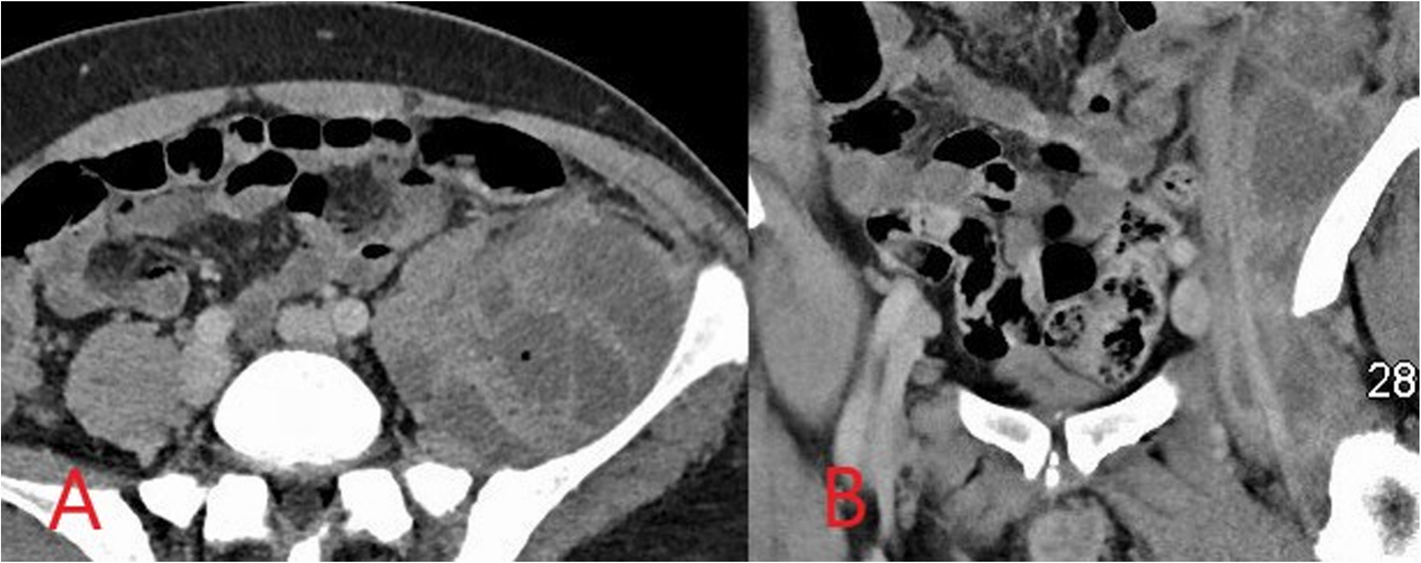
Axial computed tomography (CT) image (**a**) demonstrating a complex loculated left iliopsoas abscess in an intravenous drug user. Coronal CT image (**b**) shows extension from the left groin where the infection began due to non-sterile drug injection. Repeated CT-guided drainage was required in addition to long-course antibiotic therapy

CT is required to assess for suspected abscess formation in deeper locations where ultrasound assessment is limited such as the retroperitoneum or deep pelvis [11]. In PWID, abscesses may develop in deep locations due to direct extension from the site of injection, such as from the groin into the pelvis or retroperitoneum, or as a result of haematogenous seeding of distant locations. The risk of abscess formation in association with soft tissue infection is increased in immunocompromised patients or by the presence of a retained foreign body, meaning PWID are often at an increased risk [6, 7]. On CT, abscesses appear as a well-defined collection with internal fluid density and a peripheral rim-enhancing pseudocapsule (Fig. 3) [8]. CT can also be used for imaging-guided abscess aspiration or drainage of deep abscesses or collections with internal locules of air which can obscure ultrasound guidance [10].

**Fig. 4:**
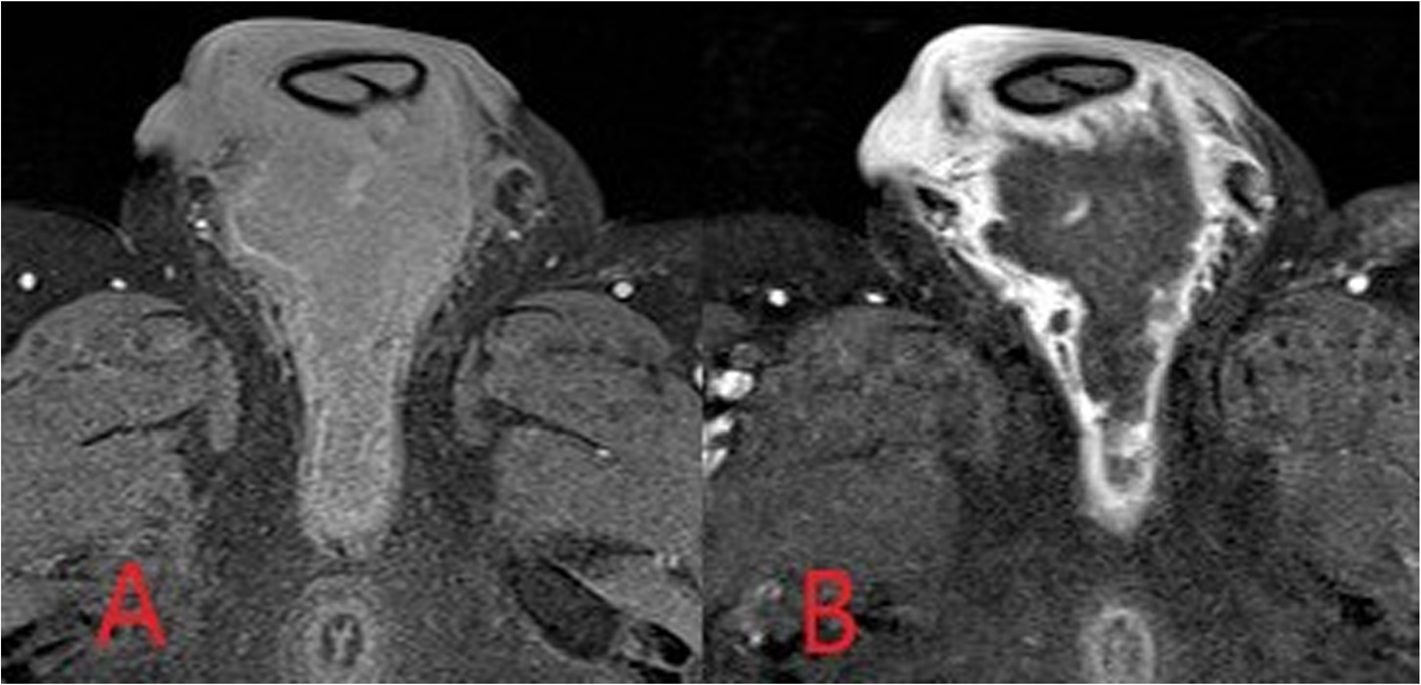
Axial fat-saturated T1-weighted MR images pre-contrast (**a**) and post-intravenous contrast (**b**) administration. An intermediate/low intensity defined collection is demonstrated within the perineum. Avid peripheral mural rim-enhancement is seen with non-enhancing internal abscess contents

If required, MRI provides the most accurate evaluation of the extent of deeper infections and of surrounding soft tissue anatomy and can also assess for associated abnormality of adjacent bones or joints [8, 12]. On MRI, soft tissue abscesses demonstrate internal high T2 and STIR signal with corresponding low T1 signal, peripherally enhancement following intravenous gadolinium administration and restrict diffusion on DWI (Fig. 4) [12].

**Fig. 5:**
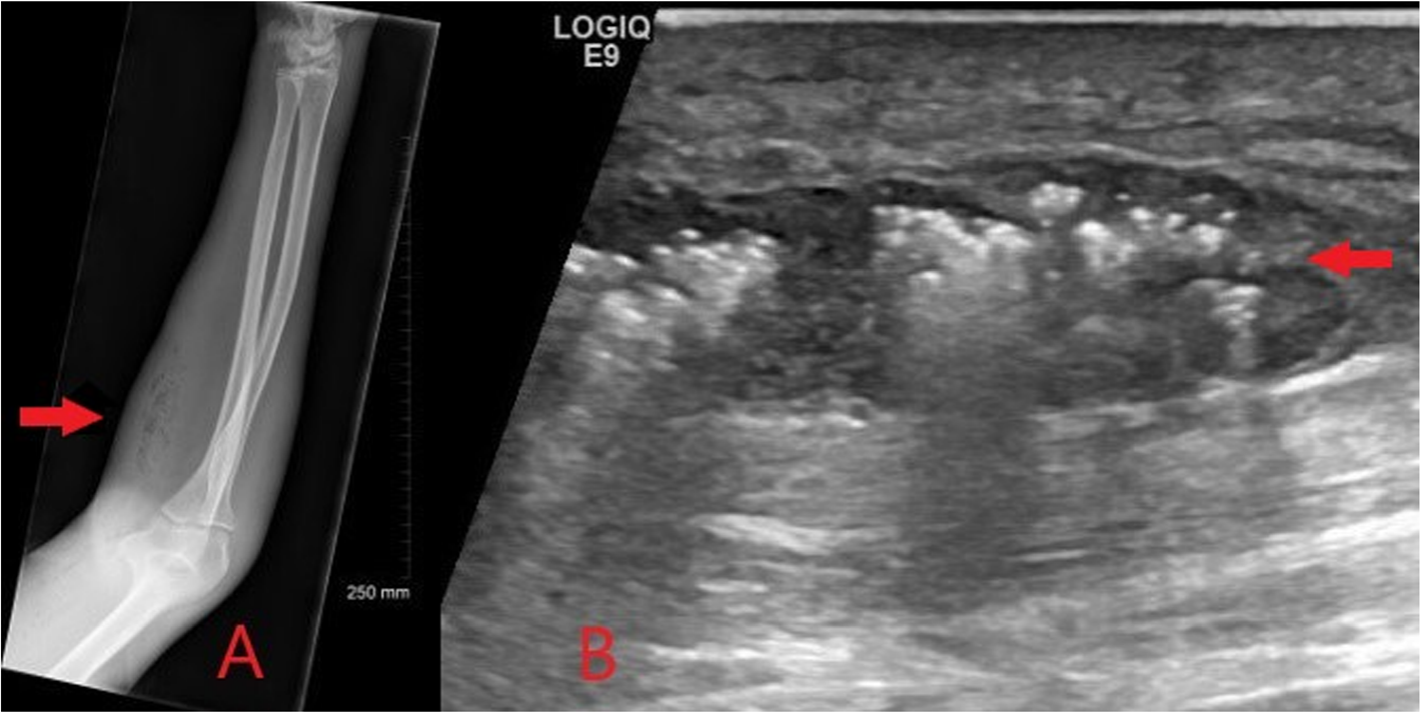
Plain radiograph (**a**) and ultrasound image (**b**) of an intravenous drug user presenting with severe forearm pain and erythema. Gas within the subcutaneous tissues is demonstrated on plain radiograph as lucency within the soft tissues (red arrow). On ultrasound a subcutaneous soft tissue abscess with internal gas artefact (red arrow) and overlying changes of cellulitis is seen. Appearances are concerning for necrotising fasciitis and prompt surgical debridement was advised

Necrotising fasciitis is a progressive, rapidly spreading infection of the deep fascia, fat and muscle with resultant secondary necrosis of subcutaneous soft tissues and carries a significant mortality rate [9]. Although it is relatively rare, prevalence is higher in those who are immunocompromised, such as in HIV patients, and in PWID. Gas-forming anaerobic bacteria are commonly involved in combination with aerobic gram-negative bacteria, and prompt surgical debridement and broad-spectrum antimicrobial therapy is necessary [13]. It is a life-threatening surgical emergency but can be difficult to recognise clinically in its early stages. Early imaging findings are similar to those in cellulitis but are more extensive and involve deeper structures. Although it is not seen in all cases, a distinguishing sign is the presence of gas within the subcutaneous tissues. This may be seen on plain X-rays as lucency within the soft tissues, or on ultrasound as hyperechoic foci with intense posterior shadowing (Fig. 5).

**Fig. 6:**
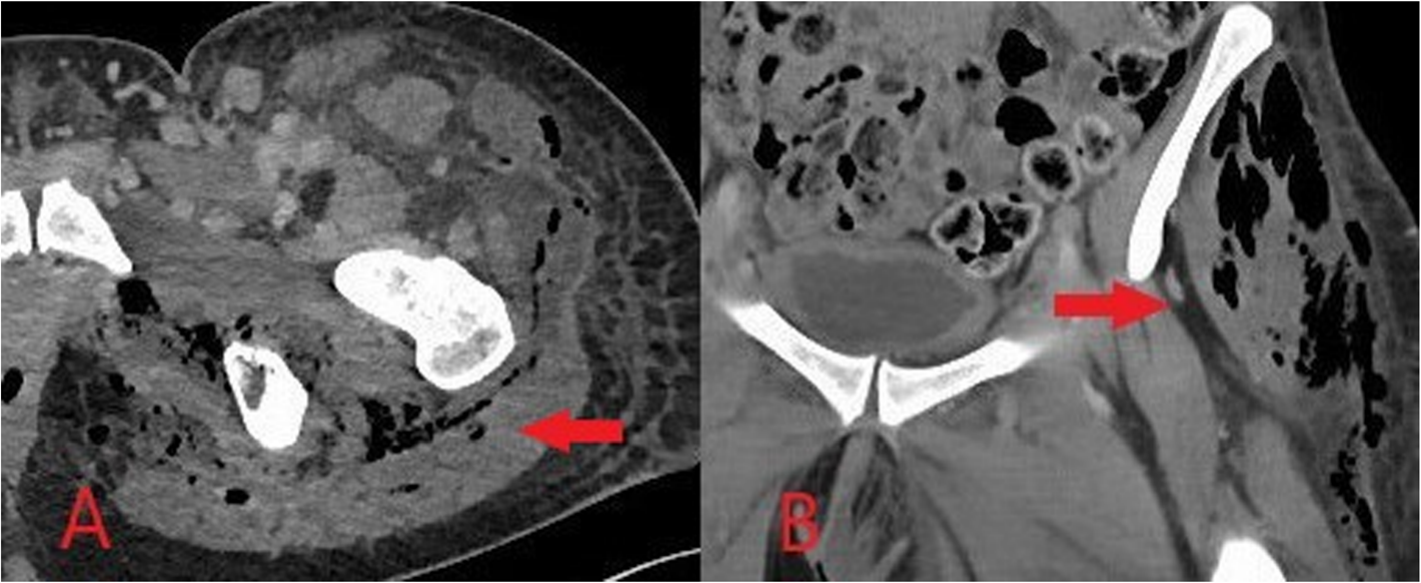
Axial (**a**) and coronal (**b**) CT images demonstrating deep fascial thickening with associated fluid and gas within the deep soft tissues in keeping with necrotising fasciitis (red arrow)

In suspected cases of necrotising fasciitis, cross-sectional imaging is often required to characterise the severity and extent of disease for surgical planning. Deep fascial thickening, fluid collections along facial sheaths and associated inflammation in adjacent fat and muscles may be seen on CT or MRI (Fig. 6) [8, 12]. There is variable post-contrast enhancement, and failure of fascia to enhance can confirm the presence of necrosis [8, 12]. On MRI, fascial thickening and oedema and any associated fluid collections are best demonstrated as increased signal intensity on T2 and STIR, and there may be loss of normal muscle texture on T1 imaging [8, 12]. Fluid-sensitive sequences such as T2 and STIR may, however, overestimate the extent of disease as a result of surrounding reactive oedema and should be interpreted in conjunction with post-contrast imaging [12]. Gas within the tissues results in MR signal loss on gradient echo sequences; however, CT is usually superior in assessing the distribution of soft tissue gas if present [8, 12].

**Fig. 7:**
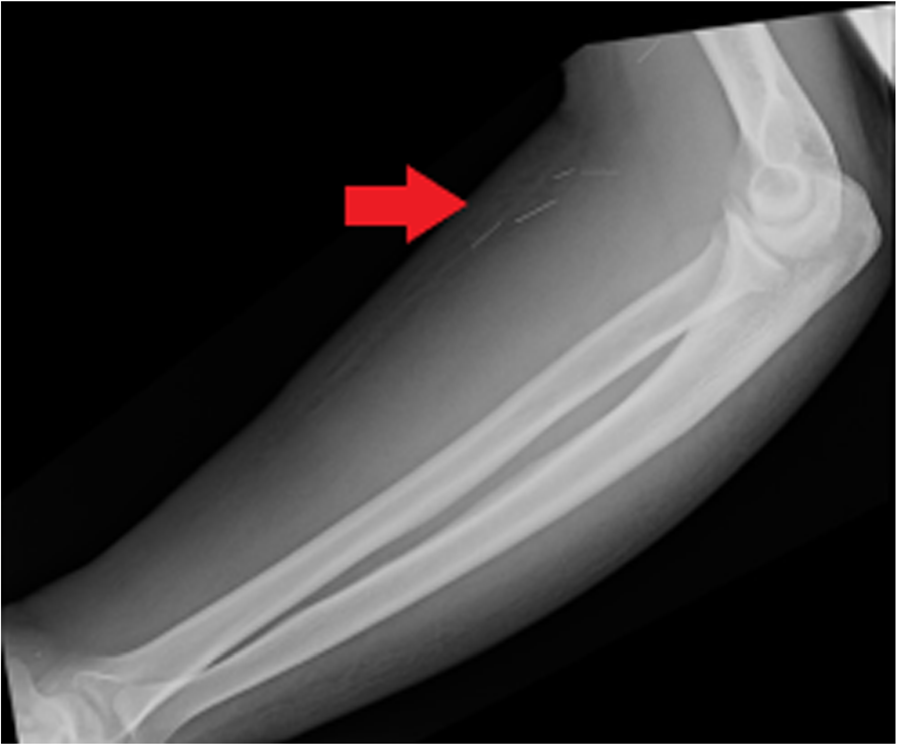
Plain radiograph of the forearm of an active intravenous drug user who presented with pain and erythema. Multiple linear radio-opaque foreign bodies representing retained needles (red arrow) are demonstrated within the volar soft tissues of the forearm

**Fig. 8:**
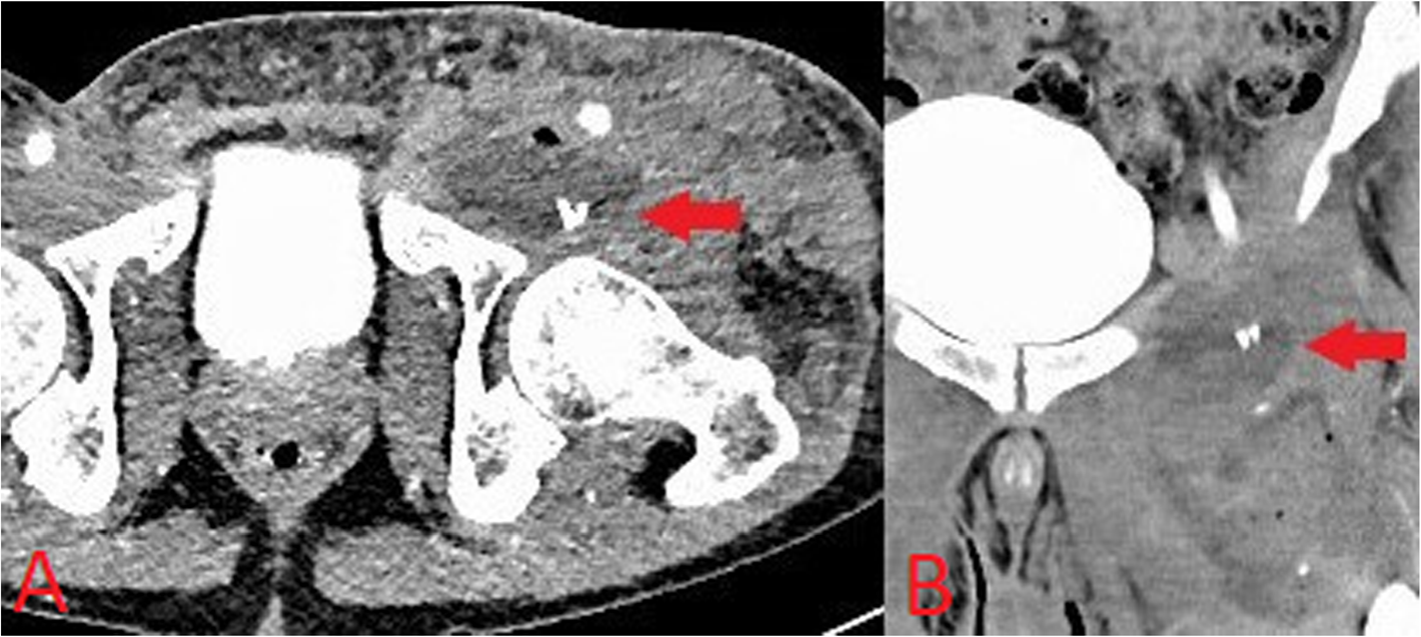
Axial (**a**) and coronal (**b**) CT images demonstrating hyper-attenuating linear structures (red arrow) associated with a groin abscess in an intravenous drug user consistent with retained needles serving as a nidus for abscess formation

An additional and often under-recognised soft tissue complication in active intravenous drug users is retained needles. Commonly, these retained foreign bodies serve as a nidus for infection and can also present a risk of needle-stick injury to healthcare workers during invasive procedures such as abscess washout/drainage. Central needle embolism is a rare but potentially serious complication and cases of needle embolism to distant locations such as the lungs and heart have been reported [14]. Needles are radio-opaque and may be readily identified on plain radiographs (Fig. 7) or CT (Fig. 8), further highlighting the value of obtaining X-rays in PWID who present with soft tissue infections.

### Bone and joint complications

Bone and joint complications of injected drug use are also predominantly infective in nature—osteomyelitis and septic arthritis chief among them—although there is also an anecdotal increased risk of fractures and other traumatic injuries related to risk-taking behaviours while under the influence of drugs [15]. Infective bone and joint complications in PWID may originate from direct extension of infection from injection sites in adjacent soft tissues or, more commonly, due to bacteraemia and haematogenous seeding [16]. In adults, haematogenous spread most frequently involves the spine leading to discitis and osteomyelitis. The risk of infective complications in PWID is also often increased as a result of co-existing immunosuppression due to chronic viral infections or cirrhosis. Important alternative causes of bone and joint infections to consider at the outset include recent trauma, recent surgery or joint instrumentation, or previous intervention involving insertion of fixation hardware or prostheses. Rheumatologic history is imperative as signs and symptoms may overlap considerably with acute or acute-on-chronic rheumatologic disorders.

Osteomyelitis is defined as bone inflammation caused by an infectious process and may be acute or chronic [17]. It is often difficult to diagnose in the acute setting and can rapidly progress to a destructive process leading to significant long-term disability and pain. Multimodal imaging plays a key role in facilitating prompt diagnosis and treatment, and early imaging should be performed in PWID presenting with signs or symptoms suspicious for osteomyelitis [17, 18]. Osteomyelitis due to haematogenous spread tends to result in a slow, insidious progression of symptoms whereas osteomyelitis from direct local extension presents with more pronounced and aggressive local manifestations. Given its insidious onset, primary or metastatic malignancy is an important differential for haematogenous osteomyelitis, particularly Ewing’s sarcoma in young adults [17, 19]. In adults, haematogenous osteomyelitis typically involves the spine and is rare at other sites [17]. Osteomyelitis as a result of local soft tissue infection may involve any bone, commonly affecting bones close to injection sites such as the extremities and pelvis in PWID.

**Fig. 9:**
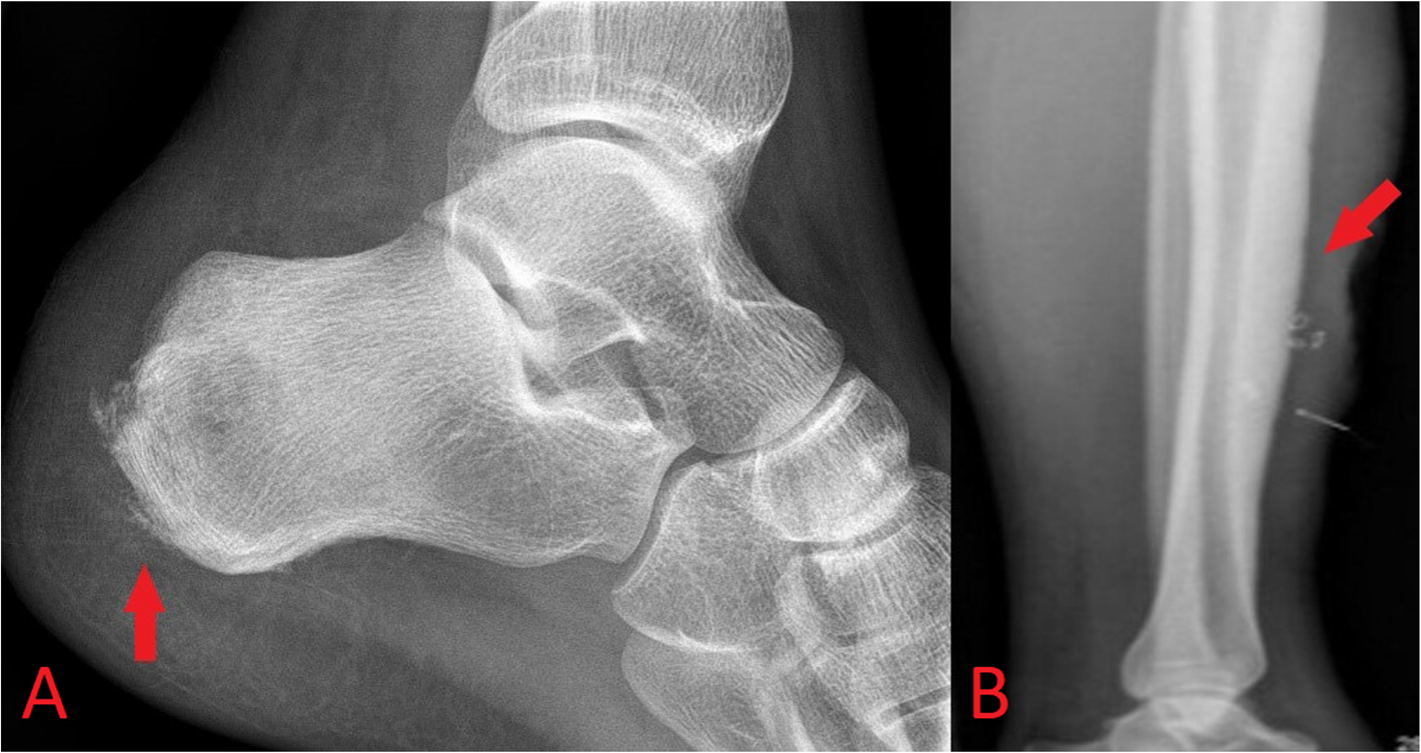
Plain lateral radiograph (**a**) of the ankle demonstrating cortical erosion of the posterior aspect of the calcaneus with associated soft tissue swelling in keeping with acute osteomyelitis (red arrow). Radiograph of the lower leg (**b**) in an ex-intravenous drug user with a non-healing ulcer anterior to the tibia shows a chronic appearing periosteal reaction along the anterior tibial border compatible with chronic osteomyelitis (red arrow)

Radiographic findings in acute osteomyelitis include periosteal reaction (Fig. 9), regional osteopenia, cortical erosion, endosteal scalloping and overlying soft tissue swelling or deformity [17, 18]. Plain radiographs are, however, relatively insensitive in early bone and joint infections as bone destruction of up to 30% may be needed for radiographic identification and visible erosions in osteomyelitis can take up to 3 weeks to develop [17, 18]. CT findings are similar to those on plain radiographs and include soft tissue swelling, regional osteopenia and cortical erosions. While CT provides greater sensitivity than plain radiographs and is the imaging modality of choice for assessment of osseous erosions, it cannot reliably identify bone oedema early in the disease process [17, 18].

**Fig. 10:**
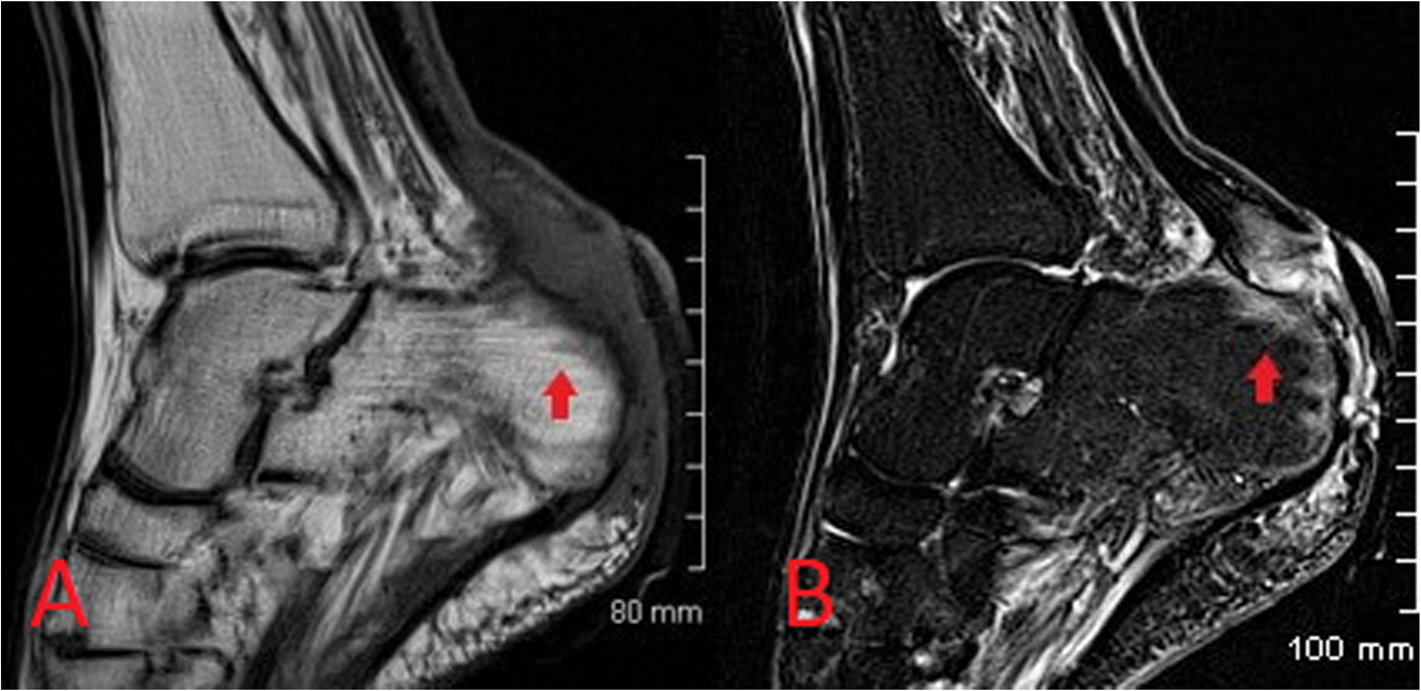
Sagittal T1-weighted (**a**) and STIR (**b**) MR images demonstrating osteomyelitis of the posterior aspect of the calcaneus. Cortical erosion and loss of the normal T1 hypointense rim at the bone edge is seen on the T1 image. There is high STIR signal and corresponding low T1 signal of the posterior calcaneus representing bone oedema (red arrow) and overlying soft tissue inflammation is also seen

**Fig. 11:**
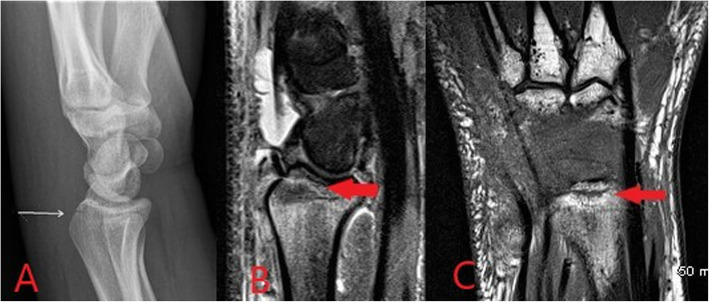
Images from an intravenous drug user presenting with wrist pain and swelling. Lateral radiograph (**a**) shows a linear lucency with cortical disruption at the dorsal aspect of the distal radius (white arrow). In the absence of a history of trauma, this was consistent with inadvertent direct injection to the bone. Associated early osteomyelitis is seen on MRI with sagittal STIR (**b**) showing a linear region of high signal with corresponding hypointense signal on the coronal T1 image (**c**)

**Fig. 12:**
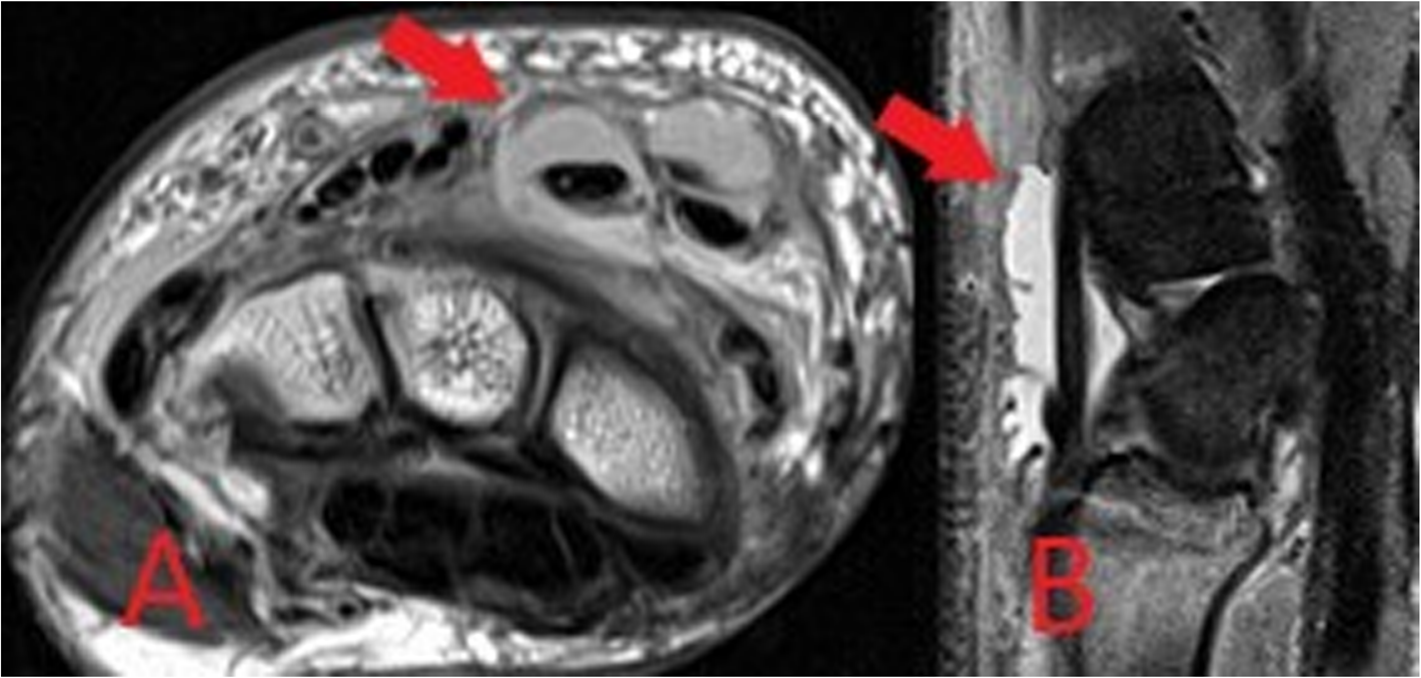
Axial proton density weighted (**a**) and sagittal STIR (**b**) MR images from the same patient as Fig. 11, demonstrating large volume fluid within the extensor compartment tendon sheaths at the wrist in keeping with septic tenosynovitis associated with injected drug use

MRI is the imaging investigation of choice in osteomyelitis and other bone and joint infections. MRI protocols for suspected bone and joint complications of injected drug use should include a fluid-sensitive sequence such as STIR in addition to T2 or proton density and T1-weighted sequences in at least two planes. Bone marrow oedema is the earliest imaging finding identifiable in acute osteomyelitis and appears on MRI as high T2 and STIR signal intensity of the bone with corresponding low T1 signal (Figs. 10 and 11) [17, 18]. Enhancement following contrast administration is also seen. High T2/STIR signal without low T1 signal is less specific and may represent reactive osteitis rather than osteomyelitis [17]. Cortical bone destruction can be identified as loss of the normal peripheral T1 hypointense cortical rim. Assessment of the extent of the infection in adjacent soft tissues, differentiation of bone from soft tissue infection and surgical planning can also be reliably achieved on MRI (Fig. 12) [17, 18].

Discitis, infection in the intervertebral disc spaces of the spine, is also frequently difficult to diagnose. Signs and symptoms may be non-specific and overlap with non-infectious causes of back pain and the source of infection is often unclear [20]. It occurs in PWID due to haematogenous spread from local infections at injection sites and can lead to serious long-term sequelae where there is a delay in treatment. It is associated with infection of the adjacent vertebral endplates, and the term discitis/osteomyelitis or spondylodiscitis is commonly used. Radiographic findings such as disc space loss and minor endplate irregularity are non-specific and overlap with common degenerative changes [20]. Significant erosion of the vertebral body endplates may take weeks to manifest. CT will display vertebral end plate irregularity and erosions at an earlier stage but remains relatively insensitive in the absence of significant surrounding soft tissue inflammation or an associated collection/abscess.

**Fig. 13:**
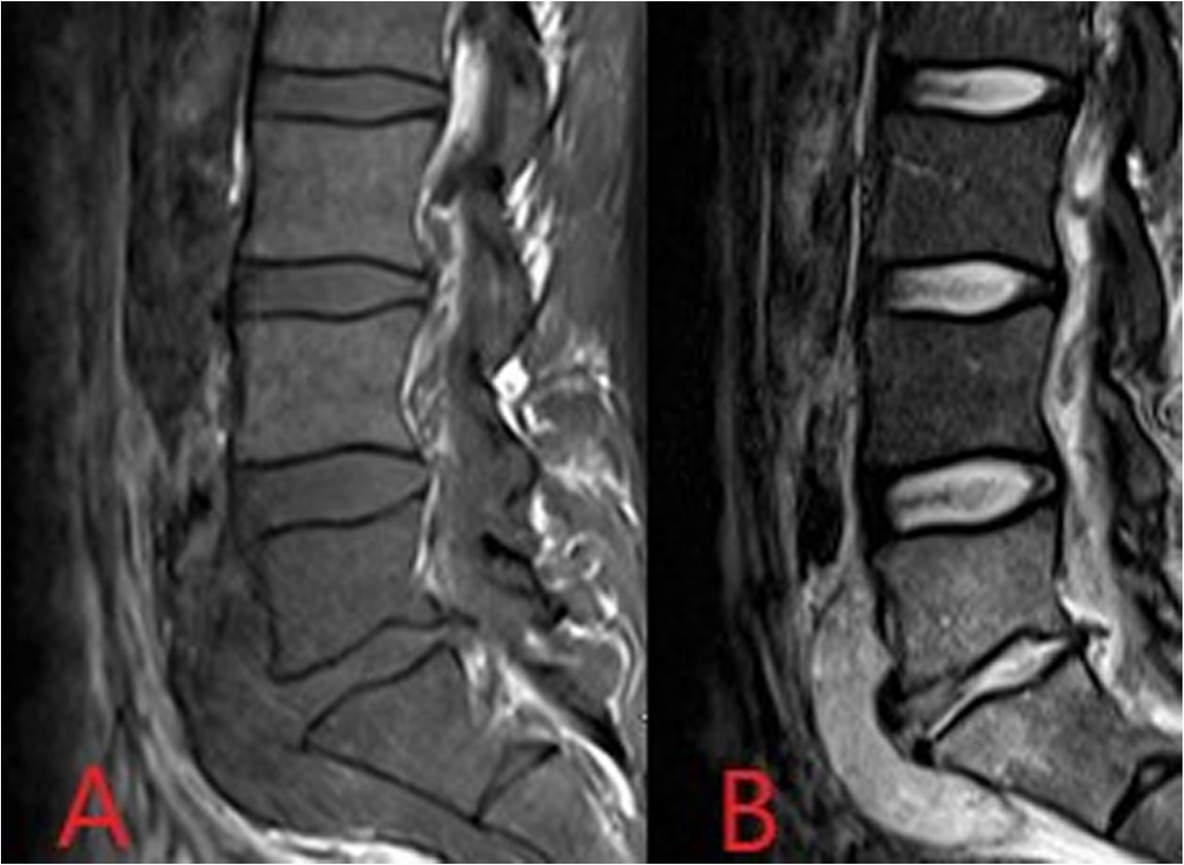
T1- (**a**) and T2- (**b**) weighted sagittal MR images demonstrating low T1 and high T2 signal within the L5/S1 intervertebral disc space with associated low T1 and high T2 signal of the adjacent vertebral bodies consistent with discitis/osteomyelitis. In addition, a paravertebral collection is seen anterior to the disc space and L5 and S1 vertebrae

MRI is, therefore, the imaging modality of choice for the acute diagnosis, similar to osteomyelitis elsewhere in the body. MRI protocols typically include sagittal T1, T2, STIR and contrast-enhanced imaging of the affected segment of the spine in addition to axial T2 and contrast-enhanced imaging at levels noted to be abnormal on initial sagittal sequences [20]. MRI demonstrates high signal intensity in the intervertebral disc space on T2 images with corresponding low signal on T1 (Fig. 13). Hyperintensity may also be seen on high B value diffusion-weighted imaging in the acute phase. Bone oedema of the adjacent vertebral body endplates also appears as high T2/STIR signal and low T1 signal. Following gadolinium administration, there is diffuse enhancement of the intervertebral disc in addition to endplate and paravertebral soft tissue enhancement. Complications such as development of an epidural or paravertebral soft tissue abscess can also be readily identified on MRI. The “imaging psoas sign”, high T2 signal within the psoas musculature, is also suggestive of discitis/osteomyelitis in suspected spinal infection [21]. An important differential to consider are Modic type 1 changes which are presumed part of the spectrum of endplate signal abnormalities seen in degenerative disease [20]. Modic type 1 changes are considered acute/subacute and also cause low T1 and high T2 endplate signal intensity. Signal within the intervening disc is typically low, however, in contrast with the high signal seen in discitis [20]. Surrounding soft tissue inflammatory change, including the “imaging psoas sign”, and clinical history indicating an increased risk of haematogenous infection, as in PWID, also suggest a diagnosis of discitis [21].

Septic arthritis usually occurs as a result of haematogenous seeding in the absence of significant trauma or recent instrumentation of the joint, though it can occur in PWID as a direct complication of local injection [22]. Large joints with a rich blood supply are typically affected by haematogenous spread, most commonly the knee [22]. In PWID, joints not classically associated with haematogenous septic arthritis may be involved such as the pubic symphysis, acromioclavicular, sternoclavicular and sacroiliac joints [11, 22, 23]. The sternoclavicular joint is particularly prone to involvement due to non-sterile injection of the upper extremity and spread along the subclavian vein [22]. Small joints of the extremities will be affected directly by local injected drug use. Prompt diagnosis and treatment is again imperative as irreversible joint damage can occur within 2 days of onset [22]. The diagnosis is typically made clinically and with direct synovial fluid sampling. Imaging is an adjunct to clinical evaluation and joint fluid analysis. It may be of particular importance in joints that are difficult to assess clinically, such as the sacroiliac joints, or where synovial fluid cannot be aspirated. In addition, imaging can assess for associated osteomyelitis in surrounding bones.

**Fig. 14:**
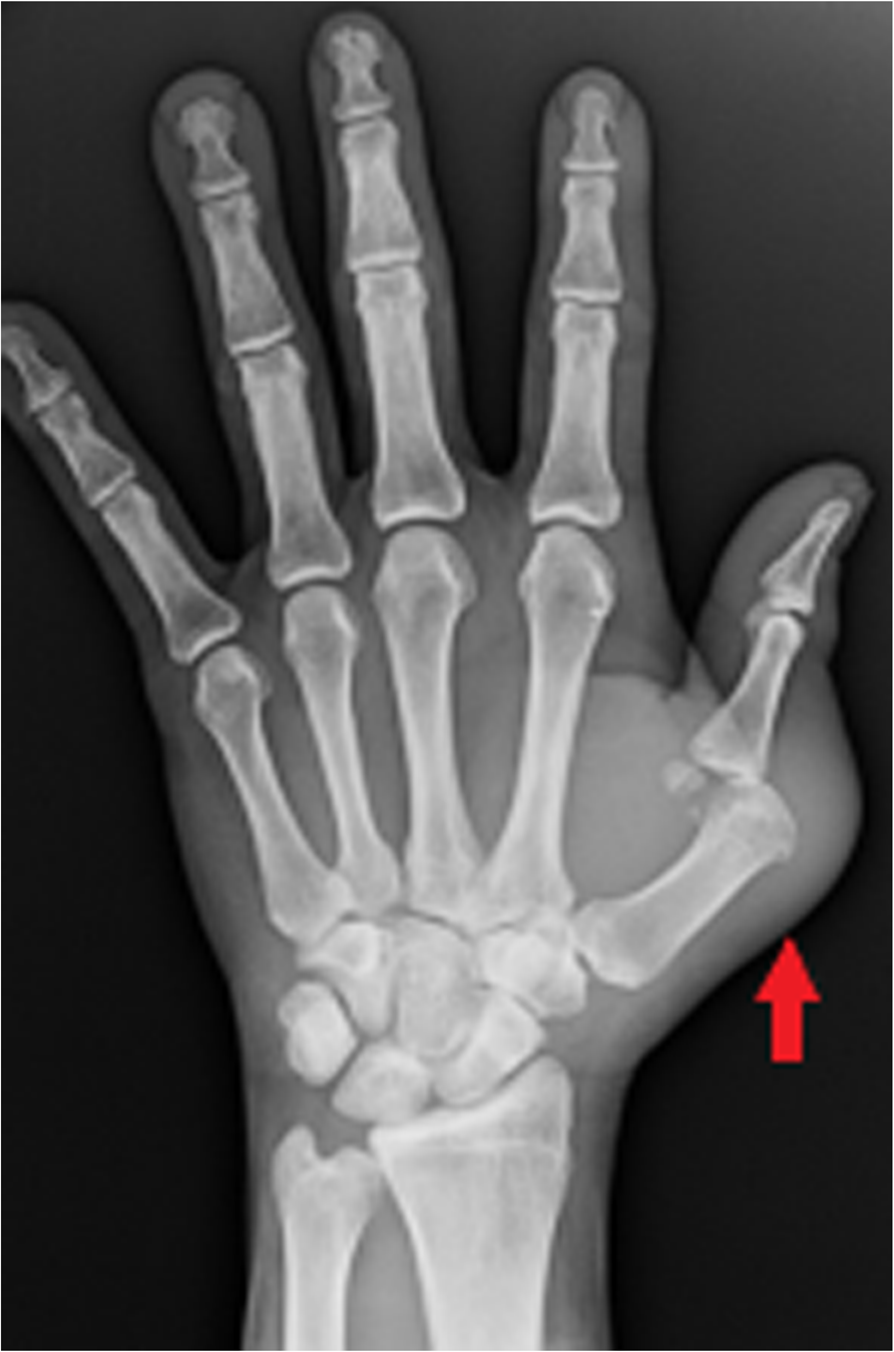
Plain radiograph of the hand in an intravenous drug user presenting with swelling, erythema and deformity of the first carpo-metacarpal joint of the left hand. A destructive and deforming arthropathy is demonstrated with peri-articular erosions, subluxation of the joint and overlying soft tissue swelling (red arrow). Joint aspiration confirmed the diagnosis of septic arthritis

Plain radiographs are the initial imaging test and can show a joint effusion and soft tissue swelling initially while peri-articular osteopenia due to local hyperaemia, joint space loss or osseous erosions may develop later (Fig. 14) [24, 25]. Radiographs cannot reliably detect effusions in many joints, however, including the hips and shoulders. CT is more sensitive for identification of early osseous erosions or intra-articular foreign bodies and may also better assess the degree of joint effusion or be used to guide joint aspiration [25]. CT is also important in the evaluation of sites such as the sacroiliac or sternoclavicular joints where plain film assessment is limited [24]. Ultrasound can be used to detect the presence of an effusion and to guide aspiration [25].

**Fig. 15:**
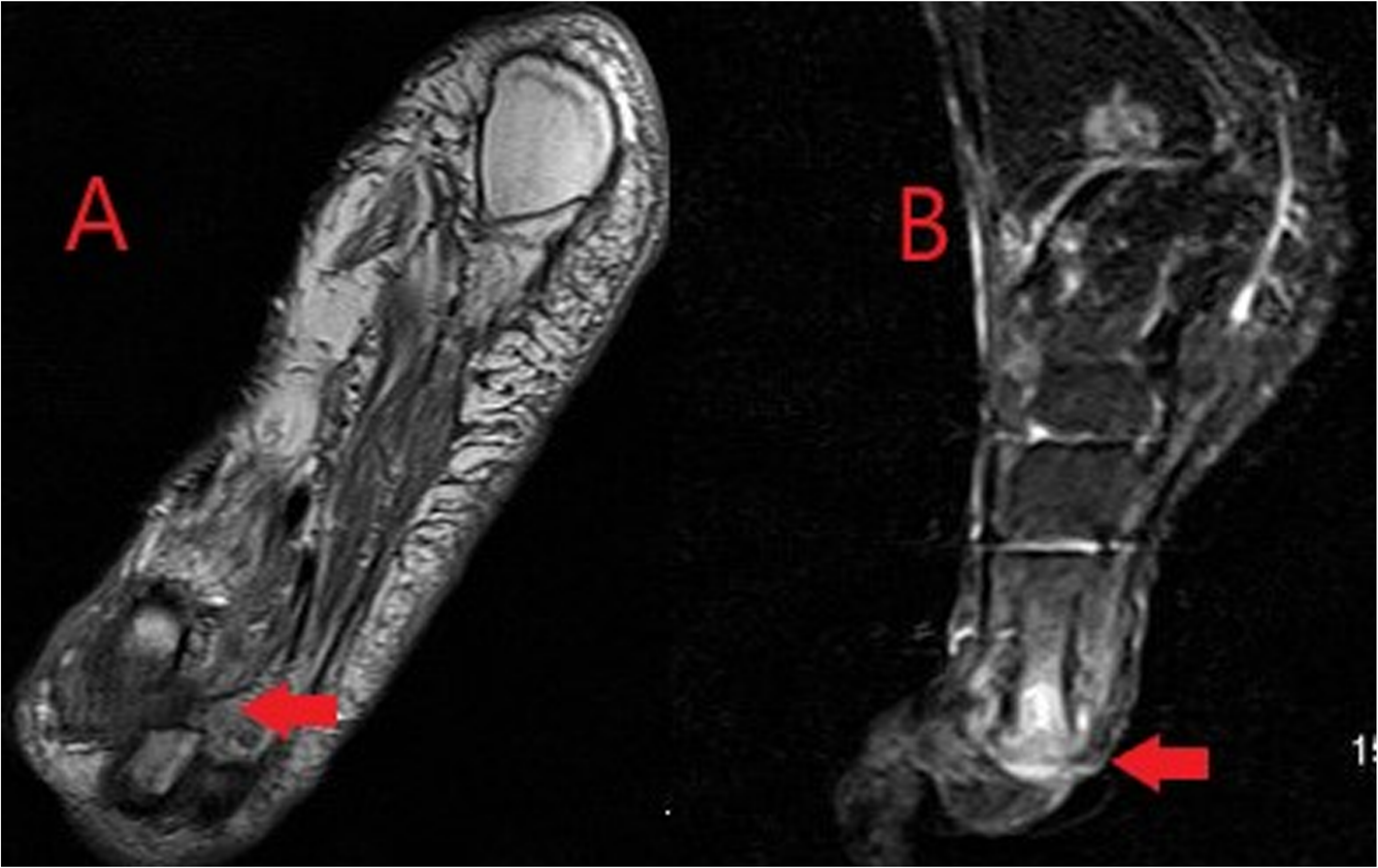
Coronal T1-weighted (**a**) and sagittal STIR (**b**) MR images demonstrating low T1 signal surrounding the first metatarsophalangeal joint of the foot with corresponding high STIR signal and joint space loss in keeping with septic arthritis and osteomyelitis (red arrows)

**Fig. 16:**
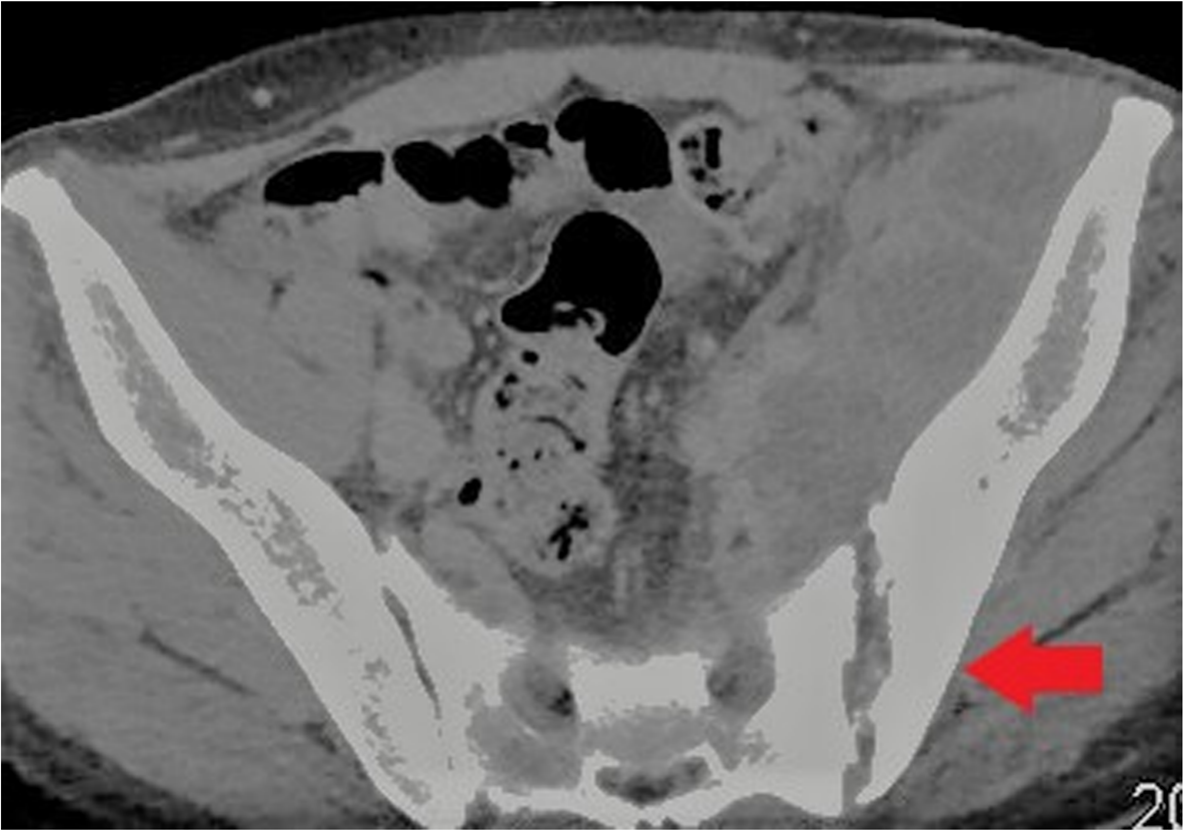
Axial CT image demonstrating chronic changes of septic arthritis at the left sacroiliac joint. Joint space widening and peri-articular bone deformity with reactive sclerosis is seen (red arrow)

Although extremely sensitive in the detection of septic arthritis, MRI findings are non-specific and can overlap with inflammatory arthrides [24]. MRI protocols typically involve T1, T2, fluid-sensitive (such as STIR) and gadolinium-enhanced sequences. MRI may be performed where there is diagnostic uncertainty or for the evaluation of surrounding soft tissues and bones. MRI will show a joint effusion with synovial thickening and enhancement, indicative of active synovitis (Fig. 15) [24]. In chronic infections, destruction of the joint surfaces and peri-articular structures occurs with resultant deformity and reactive sclerosis (Fig. 16).

### Vascular complications

Vascular complications are common in PWID, can occur locally at the injection site or at a distant location and may be arterial or venous in nature. They occur more frequently when injected drug users progress from injection of superficial upper limb veins to larger and deeper vessels such as the femoral vein in the groin or jugular vein in the neck [15, 26]. Vascular complications may manifest as injury to the vessel wall, interruption of blood flow in the lumen with resultant ischaemia or haematogenous spread of a pathogen from the injection site. Differential diagnoses which should be considered in PWID presenting with suspected vascular complications, or in patients in which a history of injected drug use is not forthcoming despite clinical suspicion, include recent trauma, recent surgery, malignancy and underlying connective tissue disorders.

Arterial complications result from inadvertent and often repeated arterial puncture during attempted venous access and can lead to acute vascular emergencies. Arterial pseudoaneurysms occur as a result of direct disruption of the vessel wall at the injection site leading to the formation of an extravascular haematoma within the surrounding tissues which retains communication with the lumen of the artery [26]. Continued communication with the high-pressure artery results in gradual expansion of the pseudoaneurysm and increasing risk of rupture. In PWID, pseudoaneurysms are frequently associated with surrounding soft tissue infection due to repeated non-sterile injection. This further increases the risk of rupture and bleeding which can be limb- or life-threatening [26, 27]. The femoral artery in the groin is the most commonly affected vessel in PWID [27].

**Fig. 17:**
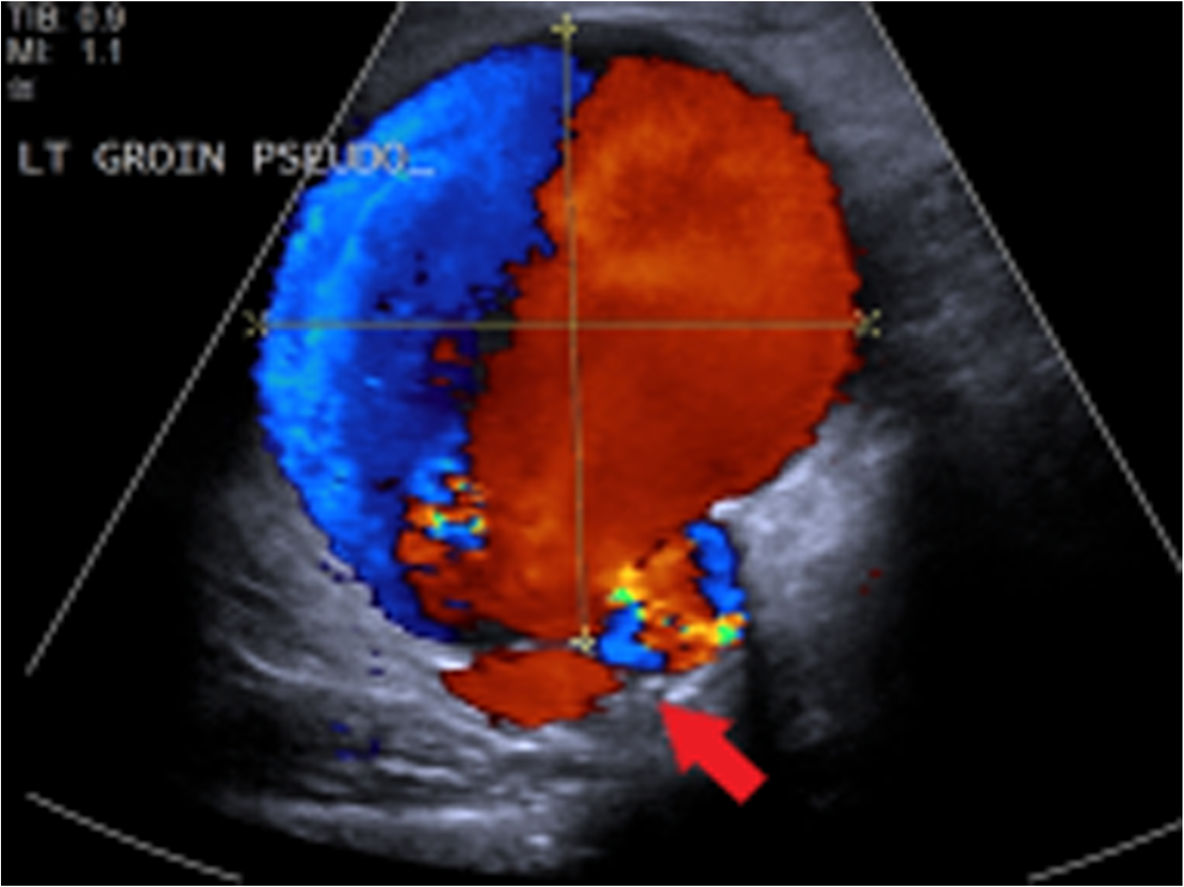
Duplex ultrasound image demonstrating a left groin pseudoaneurysm due to injected drug use. Yin-yang sign indicating turbulent pulsatile blood flow is seen within the pseudoaneurysm. The likely site of the neck communicating with the adjacent artery is also demonstrated (red arrow)

**Fig. 18:**
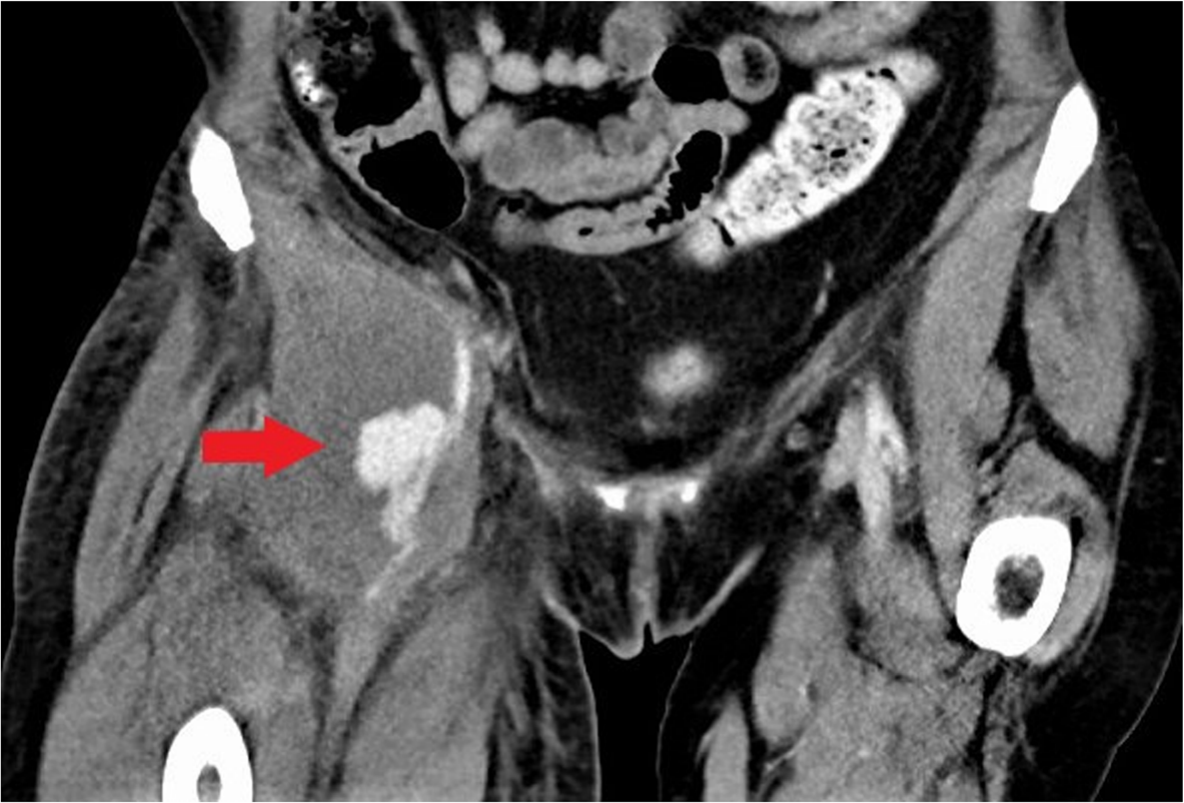
Coronal CT image demonstrating a pseudoaneurysm of the right common femoral artery (red arrow) with associated soft tissue abscess in the right groin due to intravenous drug use

**Fig. 19:**
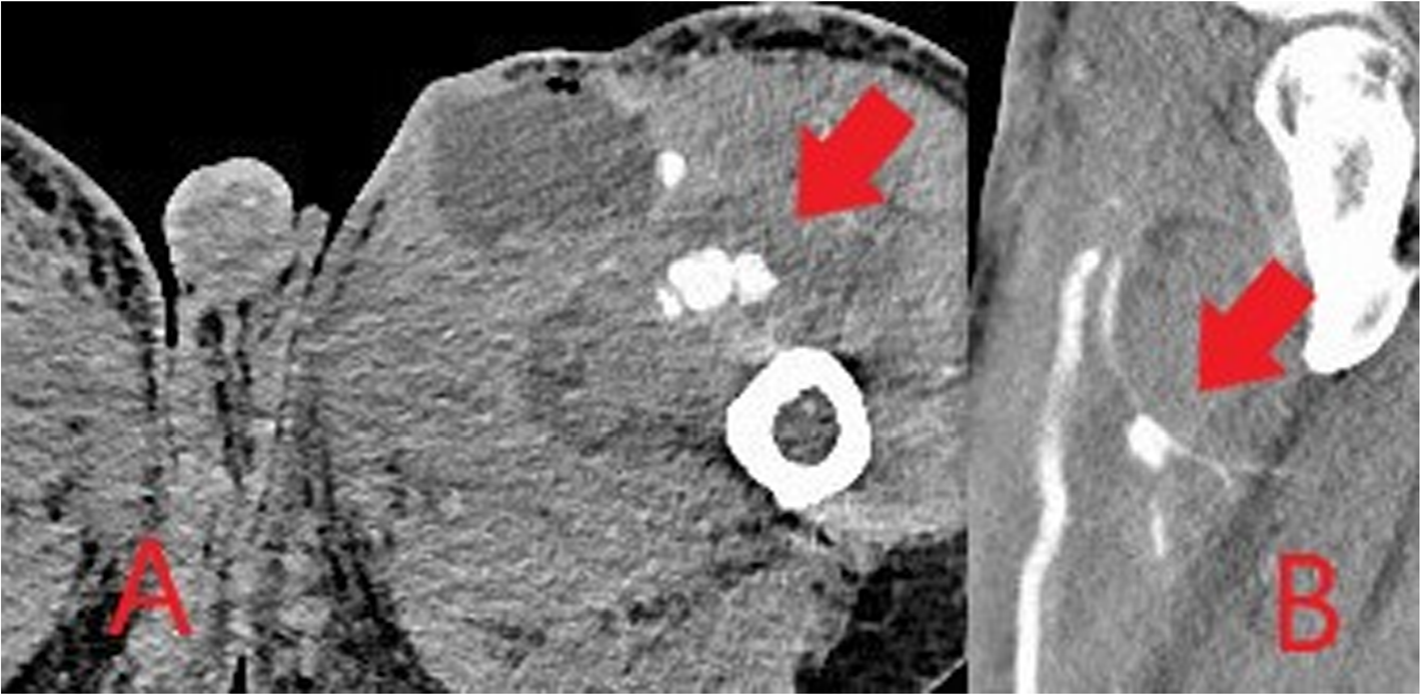
Axial (**a**) and sagittal (**b**) CT images demonstrating a pseudoaneurysm of the left profunda femoris artery (red arrow) with associated thigh/groin abscess due to intravenous drug use

Arterial pseudoaneurysms have a characteristic appearance on ultrasound which demonstrates an anechoic collection adjacent to the artery containing turbulent flow which appears as the “yin-yang sign” (Fig. 17) on Doppler ultrasound [28]. The neck of the pseudoaneurysm communicating with the adjacent artery should be identified on ultrasound. Potential treatment with direct thrombin injection may be performed with sonographic guidance if the neck is suitably narrow [29, 30]. Ultrasound follow-up is advised following treatment with thrombin injection in pseudoaneurysms at greater risk of recurrence or complication and should be considered in PWID [30]. If thrombin injection is unsuccessful or the neck is too wide, angiographic placement of a stent or surgery may be necessary [29]. Where there is difficulty in assessing the pseudoaneurysm and its neck with ultrasound, CT or MR angiography can be performed for further evaluation (Figs. 18 and 19). CT or MRI may also be used for planning of endovascular or surgical intervention in complex cases and can concurrently assess for surrounding soft tissue infection.

True mycotic aneurysms develop when there is disruption of the arterial wall due to infectious arteritis rather than as a result of direct vessel wall trauma [31]. They may occur in PWID locally at injection sites or in distant arteries due to haematogenous seeding. The aorta is the most commonly affected vessel followed by the femoral artery [31]. The majority of cases of mycotic aneurysms occur in PWID or after invasive endovascular or surgical procedures. Important differential causes to consider in patients presenting with a suspected mycotic aneurysm include diabetes mellitus and malignancy. Similar to secondarily infected pseudoaneurysms caused by direct vessel wall injury, these are also false aneurysms and are unstable and prone to rupture. In addition to the risk of rupture, mycotic aneurysms may also lead to the development of arteriovenous fistulae or serve as a source of sepsis or septic emboli [31].

**Fig. 20:**
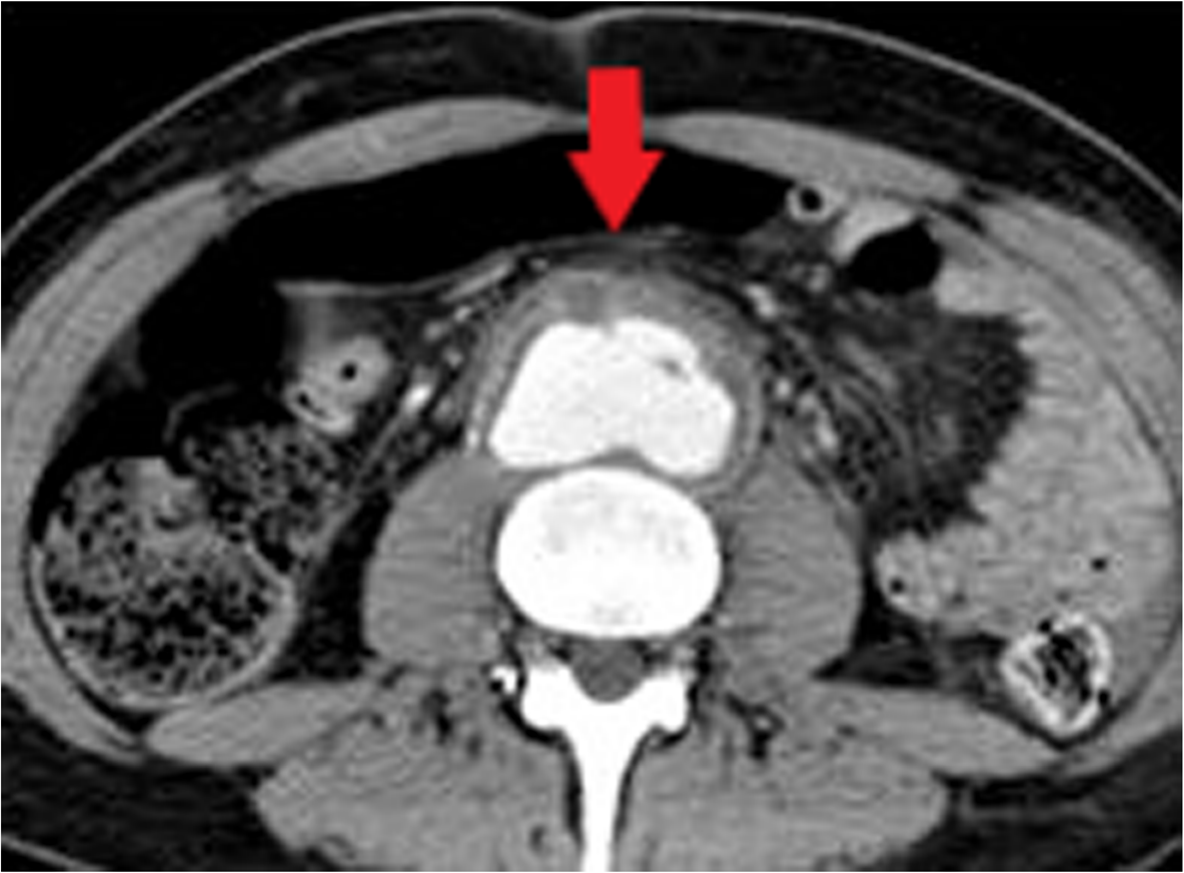
Axial CT image demonstrating a mycotic abdominal aortic aneurysm. A lobulated vascular mass with poorly defined arterial wall anteriorly (red arrow) and surrounding inflammatory fat stranding is seen

CT is the imaging modality of choice for mycotic aneurysms, although ultrasound or MR angiography may also be used for evaluation depending on the location [31]. Gas within the aneurysm is a rare but characteristic sign which is best seen on CT. Additional imaging features more commonly seen on CT include a lobulated vascular mass, an irregular and poorly defined arterial wall and peri-aneurysmal soft tissue stranding and oedema (Fig. 20). Inflammatory soft tissue surrounding the artery can develop a mass-like appearance and be associated with necrosis [31]. On ultrasound, inflammatory soft tissue is seen as a heterogenous rim of variable echogenicity surrounding the artery, though ultrasound cannot definitively differentiate infected and non-infected aneurysms [31]. On MRI, peri-arterial soft tissue oedema demonstrates low T1 signal and high T2 signal and inflammatory tissue will enhance following administration of gadolinium, except where necrosis is present. Fat suppressed T1-weighted imaging allows more detailed assessment of the vessel wall and surrounding tissues [31].

**Fig. 21:**
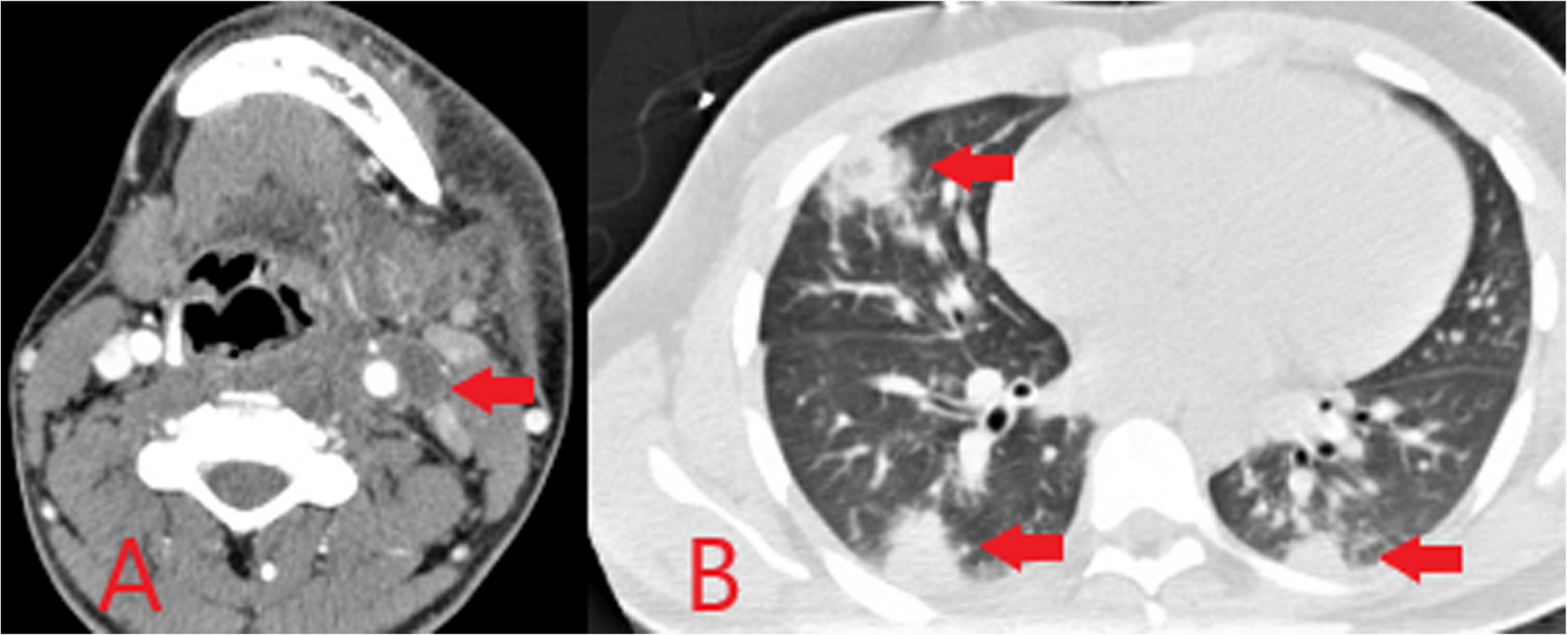
Axial CT image of the neck (**a**) demonstrating thrombus within the left internal jugular vein (red arrow) with enhancement of the vessel wall and surrounding inflammatory change consistent with thrombophlebitis following heroin injection into the neck. Axial CT image of thorax (**b**) in the same patient shows multiple rounded peripheral pulmonary opacities representing septic pulmonary emboli (red arrows)

Venous complications, particularly deep venous thrombosis (DVT), are common in intravenous drug use. Most PWID will have some degree of chronic non-occlusive thrombus at injection sites with the potential for the development and variable propagation of acute thrombus in association with this [15]. Non-sterile injection methods lead to associated infection and septic thrombophlebitis. Ultrasound is the mainstay of imaging in DVT and may also identify surrounding inflammatory changes confirming the presence of thrombophlebitis. Loss of normal vessel compressibility is the most important sonographic finding in acute DVT. Echogenic material within the vein lumen and loss of normal colour flow on Doppler can also be seen. CT can be used for further evaluation where necessary, for example in assessing for proximal extension of a lower limb DVT into the abdomen/pelvis. Both conventional and septic pulmonary emboli may occur as a complication of deep venous thrombosis (Fig. 21).

## Conclusion

Recreational drug use continues to be a significant healthcare problem and is associated with myriad multisystem complications. Musculoskeletal and vascular complications are commonly seen and are particularly prevalent in injected drug use. Awareness of the imaging manifestations and timely diagnosis of the complications related to injected drug use is important in daily radiology practice as clinical presentation may be non-specific and the history of illicit drug use often not forthcoming. A focused multimodal imaging approach is typically required, depending on the nature of suspected complications.

## Data Availability

Not applicable

## Abbreviations

AIDS: Acquired immunodeficiency syndrome
CT: Computed tomography
DALYs: Disability-adjusted life years
DVT: Deep venous thrombosis
HIV: Human immunodeficiency virus
MRI: Magnetic resonance imaging
PWID: People who inject drugs
STIR: Short-tau inversion recovery
